# Quantum Random Number Generation Based on Multi-photon Detection

**DOI:** 10.1021/acsomega.3c04584

**Published:** 2023-09-11

**Authors:** Kanin Aungskunsiri, Sakdinan Jantarachote, Kruawan Wongpanya, Ratthasart Amarit, Pongpun Punpetch, Sarun Sumriddetchkajorn

**Affiliations:** National Electronics and Computer Technology Center, 112 Thailand Science Park, Phahonyothin Road, Khlong Nueng, Khlong Luang, Pathum Thani 12120, Thailand

## Abstract

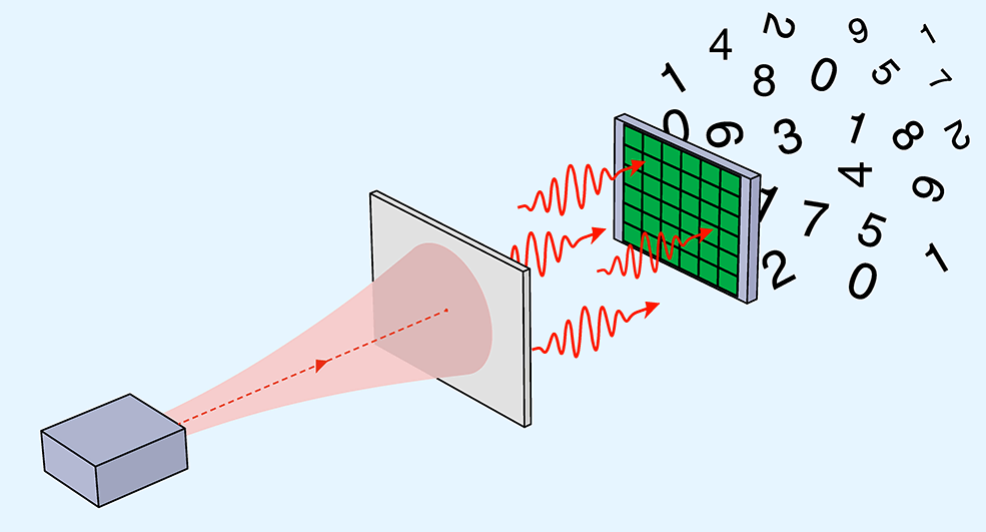

We demonstrate quantum random number generation based on a photon-number detection scheme with the use of a silicon photomultiplier. We implement a time integral with detector response signals for resolving photon numbers, which are subsequently digitized into a stream of 4-bit sequences with a generation rate of 13.6 Mbit/s. Our generated random bits pass the statistical randomness validation according to the U.S. National Institute of Standards and Technology (NIST) Special Publication 800-22. This scheme is implementable with inexpensive components, and the system can be miniaturized to the size of a plug-and-play portable cryptographic device.

## 1 Introduction

Random numbers are fundamental to a broad spectrum of modern technologies. They are indispensable in lottery winner selection, the gaming industry, scientific simulation,^1^ and encryption. They are of key importance in the process of establishing the communication links in network security protocols, e.g., the Secure Shell (SSH), the Secure Sockets Layer (SSL),^2^ and the Transport Layer Security (TLS). Modern banking necessitates random numbers to create one-time passwords that are implemented for user authentication. Upcoming quantum technologies demand true entropy sources for the generation of the secret keys used in quantum key distribution^3−5^ and blind quantum computing.^6^

As opposed to pseudorandom numbers, which are artificially produced from a computerized algorithm, truly random numbers are difficult to generate. Various natural phenomena, for instance, radioactive decay, atmospheric turbulence,^7^ tunneling effect in semiconductor devices,^8−12^ and Raman scattering,^13,14^ serve as accessible sources of true randomness. Photonics is a promising platform for achieving particularly high-bitrate entropy sources for different schemes, e.g., photon branching path using a beam splitter,^15−17^ laser phase noise detection via an interferometer,^18,19^ vacuum fluctuation measured by homodyne detection,^20−25^ and photon statistics determined by either time of arrival^26,27^ or photon number distribution.^28−31^

Although true randomness and generation rate are the supreme criteria, key issues such as complexity, cost, and reliability of the system should also be considered for real-world applications. This holds true for quantum entropy sources like optical phase noise and vacuum fluctuations, which can both achieve the generation rate in the Gbit/s regime; however, implementations are technically challenging and expensive. Quantum entropy sources realized from optical phase noise necessitate a temperature control^18^ or feedback loop^19^ to create a stable interferometer, both of which introduce complexity. A scheme based on vacuum fluctuations^20−25^ is challenging for practical adoption outside laboratory environments as it requires a bulky beam splitter and a costly balanced detector for homodyne measurements. Among these implementations, a scheme based on detecting single photons in low-intensity light has the advantage of a simple optical arrangement with only an attenuated light source and a detector.

The quantum random number generation (QRNG) presented here was realized from a photon-number measurement scheme. This scheme has been demonstrated through the use of conventional^32^ and quantum image sensors.^33^ Conventional image sensors with a high pixel density are widely integrated into consumer devices; however, they are incapable of detecting single photons. The QRNG derived from conventional image sensors^32^ encounters a technical obstacle caused by the low quality of an entropy source, necessitating postprocessing with a high compression ratio. As a result, the output data rate is significantly reduced. On the other hand, quantum image sensors with the capability of single photon detection offer an alternative solution for achieving a high-quality entropy source with the potential to generate high throughput.^33^ Unfortunately, the technology of quantum image sensors is still in a developing state,^34^ given that the implementation cost of the QRNG is expected to be expensive.

Variations of QRNG realized from a photon-number measurement scheme have also been demonstrated using different photon counting technologies, including a photomultiplier tube (PMT),^28^ an avalanched photodetector (APD),^31^ and a silicon photomultiplier (SiPM).^29,30^ These days, both PMTs and APDs are still expensive. The use of a PMT requires a costly high-voltage power supply. In comparison, the SiPM, commonly known as a multi-pixel photon counter, operates at low voltage and is available at an affordable unit price.

Existing realizations of QRNG that employed an SiPM for multi-photon detection^29,30^ have relied on the technique of taking the peak height of the pulse for resolving photon numbers. This photon-detection technique particularly succeeds when all of the photons arrive at the SiPM simultaneously. Therefore, this scheme demands a high-speed pulse generator and a fast-modulation laser diode, both of which are expensive, to create a pulse train of light with a very short pulse duration (less than 1 ns). This way, the peak height of the detection signal corresponds to the sum of the amplitude of concurrent detection events.

In this work, realization of our QRNG used an SiPM for multi-photon detection but with a more affordable and practical approach. Our key idea relies on a technique that implements a time integral with a detector response signal to derive the number of photons detected within a given time. Because each photon signal produces a fixed amount of charge, this approach provides a more robust measurement than taking the amplitude of the signal. Additionally, the arrival of photon flux is not restricted to sub-nanosecond pulse durations, resulting in a system that is implementable with a low-cost laser diode.

## 2 Method

**Figure 1:**
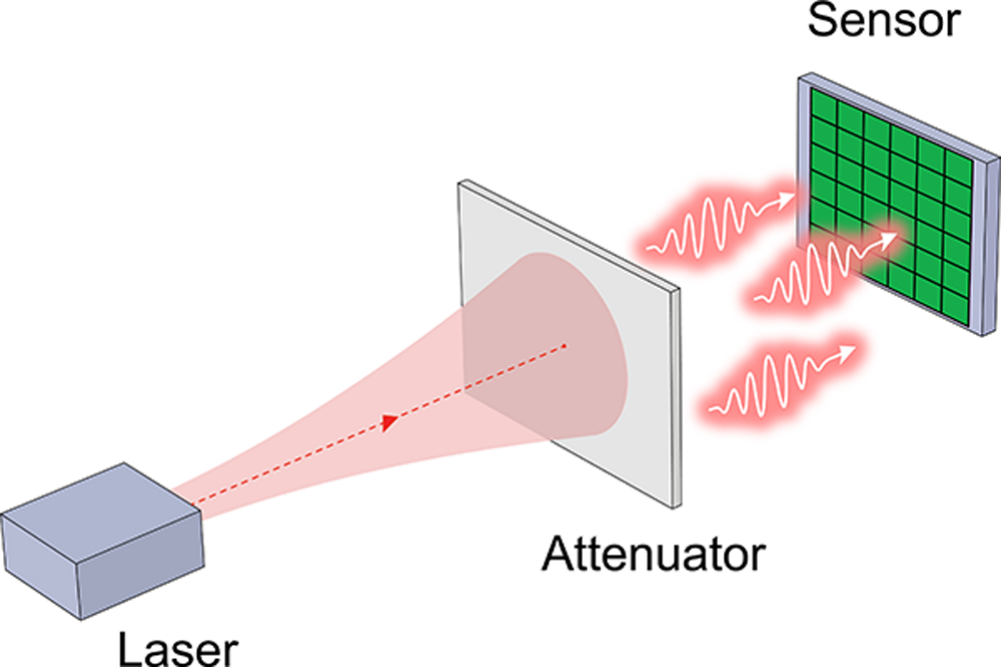
Conceptual setup for quantum random number generation. An SiPM sensor is illuminated with an attenuated photon flux. Loss due to the photon detection efficiency of the sensor can be regarded as optical attenuation.

In quantum optics, a collection of small photon flux from a laser diode that emits coherent light with a constant intensity can be statistically modeled with a Poisson process. The probability of finding *n* photons, *P*(*n*), in a constant time obeys the Poissonian distribution described as
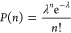
1where λ is an average number of photons per a given time. Taking advantage of this intrinsic nature of light, realization of our QRNG is based on measuring the flux of photons using an SiPM (Figure 1).

Random number generation involves the concept of min-entropy, *H*, which is a standard measure of randomness used for characterizing a noise source. This quantity indicates that the certainty of the data is no greater than 2^–*H*^. The min-entropy of a dataset with the probability distribution *p*_*j*_ for *j* = 1,2, . ., *k*, is expressed in bits and defined as
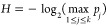
2

**Figure 2:**
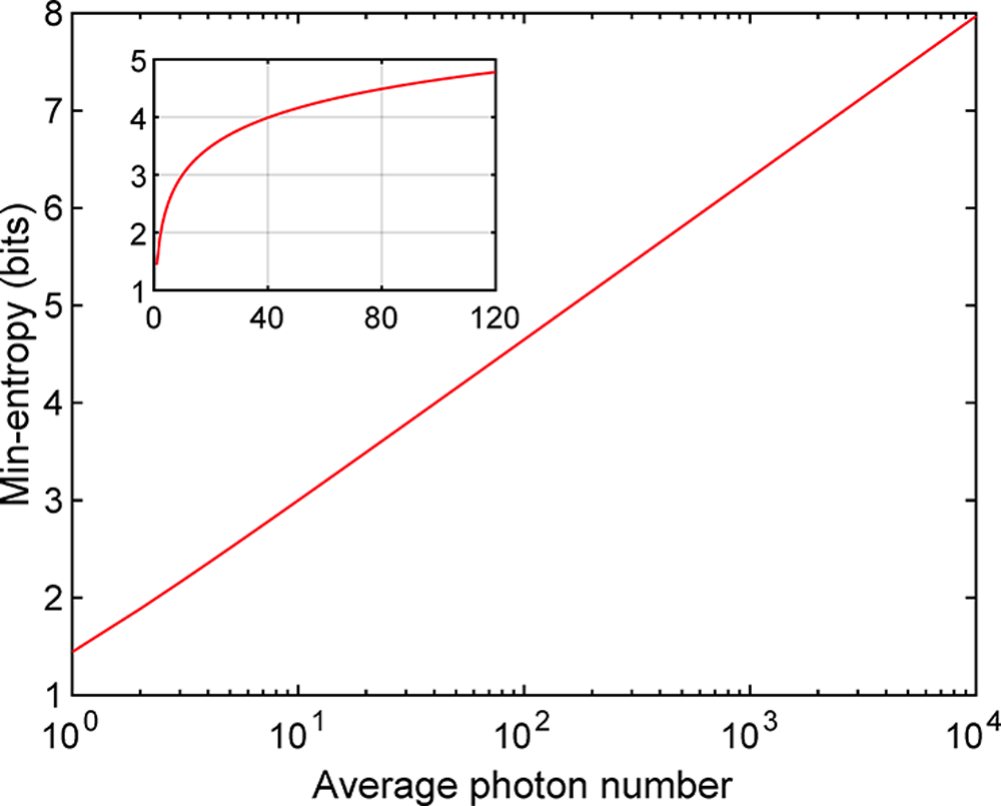
Theoretical plot of min-entropy values (vertical axis) vs average photon numbers, *N* (horizontal axis), calculated from a noise source modeled with a Poisson process. The inset presents the linear plot at small *N*.

This value corresponds to the amount of quantum entropy, indicating the upper bound for the number of random bits that can be extracted from the noise source. Taking eqs 1 and 2, we can calculate the min-entropy value of an optical noise source modeled with a Poisson process. The theoretical values of the min-entropy at different mean photon numbers are plotted in Figure 2. In the step of our QRNG realization, we will incorporate this quantum entropy estimation to determine an appropriate optical attenuation.

SiPM is a photon detector with high-count-rate and photon-number-resolving capabilities. An SiPM is made of multiple silicon APD pixels that are connected in parallel and share a common anode and cathode. Each pixel has its own quenching circuit, indicating that each individual pixel that responds to photon absorption generates an avalanche signal independently. The output obtained from an SiPM corresponds to the cumulative sum of signals generated by each individual pixel that can respond to only one photon at the same time.

**Figure 3:**
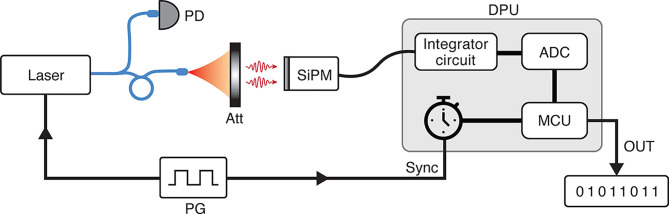
Ideal setup for realizing QRNG. Light from a fast laser diode (Osram PLT5-520EA_P) is attenuated (Att) to achieve the single-photon level before illuminating onto an SiPM-integrated detector (Hamamatsu C14455-1550GA). A pulse generator (PG, Hantek HDG6202B) is used for modulating the optical intensity. The detector response signals are sent to a data processing unit (DPU) for extraction of the random numbers. This DPU is made of a timer that is synchronized with the PG, an integrator circuit that acquires the detector response signals, an analog-to-digital converter (ADC) that calculates the time integral with the voltage signals, and a microcontroller unit (MCU) that converts a voltage-time-integral value into random numbers. A photodiode (PD) is a supplement used for monitoring the optical intensity of the light source.

Our scheme relies on the assumption that the light source and measuring devices are trusted, meaning that the setup is independent and isolated from any potential influence exerted by an adversary. Figure 3 presents an ideal setup for the realization of our QRNG. Light from a laser source is sent through an optical fiber to illuminate a fast photodetector (PD) and an SiPM-integrated detector. The laser intensity is attenuated (Att) to achieve a small photon flux before it arrives at the SiPM. A pulse generator (PG) is used for intensity modulation of the light source. Response signals from the SiPM are sent to the data processing unit (DPU) for conversion into random numbers. This DPU contains an integrator circuit for acquiring the SiPM’s response signals, a timer that is in sync with the PG, an analog-to-digital converter (ADC) that converts an input signal into a voltage-time-integral value, and a microcontroller unit (MCU) that subsequently converts the value into a photon-detection number and then random numbers. This setup is implementable with inexpensive off-the-shelf components. In practice, a field programmable gate array (FPGA) can serve as a replacement for the PG, timer, and MCU. To prove the concept of our QRNG, we employed an oscilloscope to collect the detector response signals and then processed the data on a computer.

**Figure 4:**
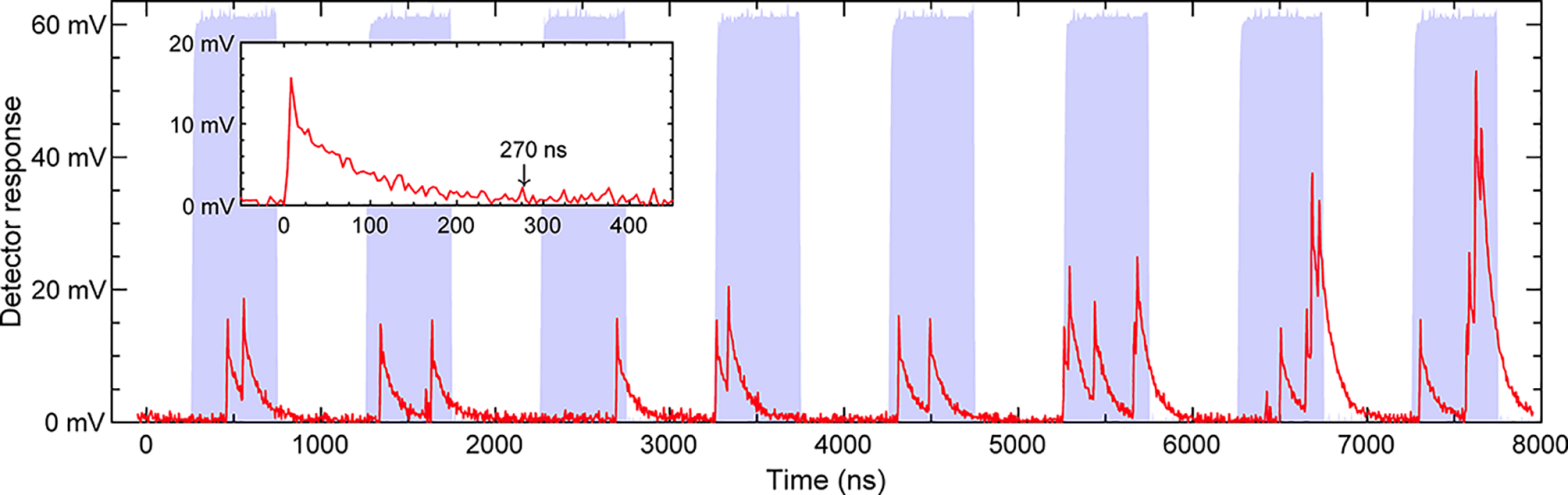
Time-series plots of the optical intensity of the light source (blue area) and SiPM’s response signals (red line). The inset presents the response signal from an event of one photon detection. Optical intensity was monitored with a fast photodiode (Thorlabs PDA8A2), and its pulse height was normalized for ease of illustration. Data were collected with an oscilloscope (Hantek 6254EU) with a time resolution of 0.2 ns and a voltage resolution of 8 mV.

To characterize the response signals of the SiPM, the light source was modulated to emit a pulse train of 500 ns width with a repetition rate of 1 MHz. We denote the duration when light is on and off in one modulation cycle as the pulse active time (light period) and the idle duration (dark period), respectively. The optical attenuation was adjusted to achieve less than five photon detections per pulse. The results are plotted in Figure 4. As the figure shows, signals of photon detection initiated within the pulse active time, except for signals from dark counts that may occur at random times. Photon detections near the edge of the pulse active time provided a signal that spans across the idle duration. With the occurrence of multi-photon detection, the signals produced from multiple pixels are piled up to form the response signal. A photon detection, as plotted in the inset, produced a response signal with an approximated pulse width of 270 ns. According to the data, a voltage-time-integral value associated with an event of one photon detection, including an offset, can be calculated.

The QRNG realized here was specifically based on a photon-number measurement scheme that calculates a photon number from voltage-time integration of a response signal for a period of one modulation cycle. It is essential that each integration window covers the full width of occurring response pulses, yielding that the minimum width of the idle duration is restricted to the pulse width of one photon detection, which is 270 ns for our SiPM. The pulse active time was determined from the modulation speed of the light source, which is 100 MHz for our laser diode. Based on these constraints, the laser source was modulated to produce a pulse train of 20 ns width with a repetition rate of 3.4 MHz, which almost reached the maximum data acquisition rate achievable with this setup.

In practice, false detections may arise as a result of dark counts, crosstalk, and afterpulsing processes, leading to an overestimation of the quantum entropy. The total detector response (*N*_tot_) is the sum of responses from a photon flux (*N*_photon_), dark counts (*N*_dc_), crosstalk (*N*_ct_), and afterpulsing events (*N*_af_), such that
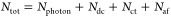
3

During the operation of the QRNG, we can measure only *N*_tot_, leaving the other values unknown. Knowing *N*_photon_ provides the necessary information for the estimation of quantum entropy according to eq 2. Since *N*_photon_ is not directly measurable, we then implement the following procedure to estimate the quantum entropy.

We assume that the noises from dark counts, crosstalk, and afterpulsing events are Poisson processes.^35−37^ Since the sum of Poisson processes is also a Poisson process, the total detector response is described by a Poisson process. Accordingly, the mean value of the detector response (μ_tot_) is the linear sum of mean numbers of photon detection (μ_photon_), dark counts (μ_dc_), crosstalk (μ_ct_), and afterpulsing (μ_af_) events, such that
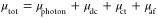
4

The occurrences of crosstalk and afterpulsing events are proportional to the rate of photon detection, with probabilities ρ_ct_ and ρ_af_, respectively. Hence, we can rewrite eq 3 as
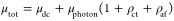
5

Based on the data acquisition rate of our QRNG, which is 3.4 MHz, and given the known parameters of our SiPM, we obtained μ_dc_ = 0.016, ρ_ct_ = 0.05, and ρ_af_ = 0.001. Therefore, we can estimate
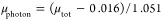
6

Since our setup has μ_photon_/μ_tot_ ≈ 1, we can approximate the total detector response as the number of photon detections.

Quantum entropy relies on the statistical distribution of *N*_photon_, which follows a Poisson distribution. The obtained value of μ_photon_ is the key for calculating the min-entropy value. By substituting λ = μ_photon_ into eq 1 to find the peak value of the distribution and incorporating eq 2, the min-entropy value can be estimated.

**Figure 5:**
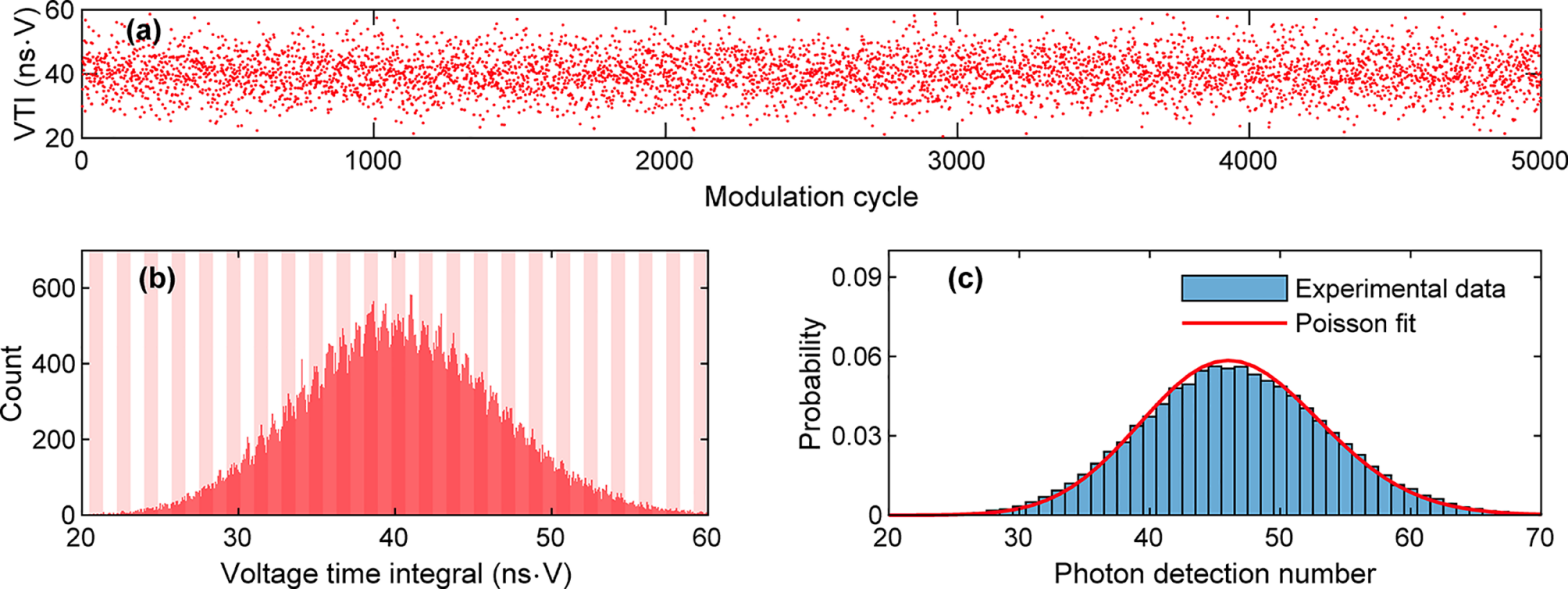
Experimental data showing (a) an example of voltage-time-integral (VTI) values obtained from 5,000 modulation cycles, (b) a histogram of VTI values collected for 100,000 modulation cycles, and (c) a histogram of the associated photon-detection number versus a Poisson model plot (red solid line) with a mean value of 46.5364. Data were collected with a time resolution of 0.2 ns and a voltage resolution of 3.937 mV, resulting in data acquisition having a resolution of δ = 0.7874 ns·mV. In (b), the histogram has a bin size of 100δ, and each vertical block represents a bin size of one photon. In (c), the histogram has a bin size of 1.

Next, we consider an appropriate optical attenuation for the implementation. Theoretically,^38^ photon detection deviates from linearity by 5% when the number of impinging photons is at 10% of the total number of pixels. Since our SiPM has 720 pixels, it is necessary to limit the impinging photon flux on a sensor to well below 72 photon detections per pulse to avoid the effects of a non-linear response of the detector. We incorporated the entropy estimation, as presented in Figure 2 for determining a suitable photon-detection number. By configuring the optical attenuation to achieve approximately 45 photon detections per modulation cycle, the quantum entropy would exceed 4 bits. The statistics of the experimental data are presented in Figure 5.

Figure 5a presents an example of data calculated from voltage-time integration. A statistical distribution of the experimental data is presented in Figure 5b. These data were then discretized into photon-detection numbers and presented in Figure 5c. The statistical distribution, as presented in Figure 5c, has a mean value of μ_tot_ = 46.5364 with a standard deviation of σ_tot_ = 7.1414. We calculated the Fano factor, *F* = σ^2^/μ, and obtained *F*_tot_ = 1.0959. This value is close to one, suggesting that the experimental data can be reasonably approximated by a Poisson process. By substituting the value of μ_tot_ into eq 6, we obtain μ_photon_ = 44.2630.

## 3 Random Bit Extraction

Now, we adopt the concept of min-entropy presented earlier in eq 2 for random bit extraction. By substituting λ = 44.2630 into eq 1 and incorporating eq 2, we obtained a min-entropy of 4.0593 bits. This value indicates the maximum bit length extractable from a single event. Accordingly, data collected from each event will be rendered to the stream of 4-bit sequences. We implemented a method that calculates the second-order derivative for multi-bit extraction. This method does not require computation complexity and can be implemented in real time using an MCU. Taking time-series data of photon-detection numbers, *y*_*i*_, for *i* = 1,2,..., *n*, the second derivative, *ÿ*_*i*_, was calculated from
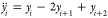
7

Each value of *ÿ*_*i*_ was converted into an integer *X*, for *X*∈ [0,15], such that
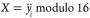
8

Finally, *X* was then digitized into 4-bit sequences as binary outputs. As our QRNG was realized with data acquisition at a rate of 3.4 MHz, the generation rate of the binary outputs was 13.6 Mbit/s.

**Figure 6:**
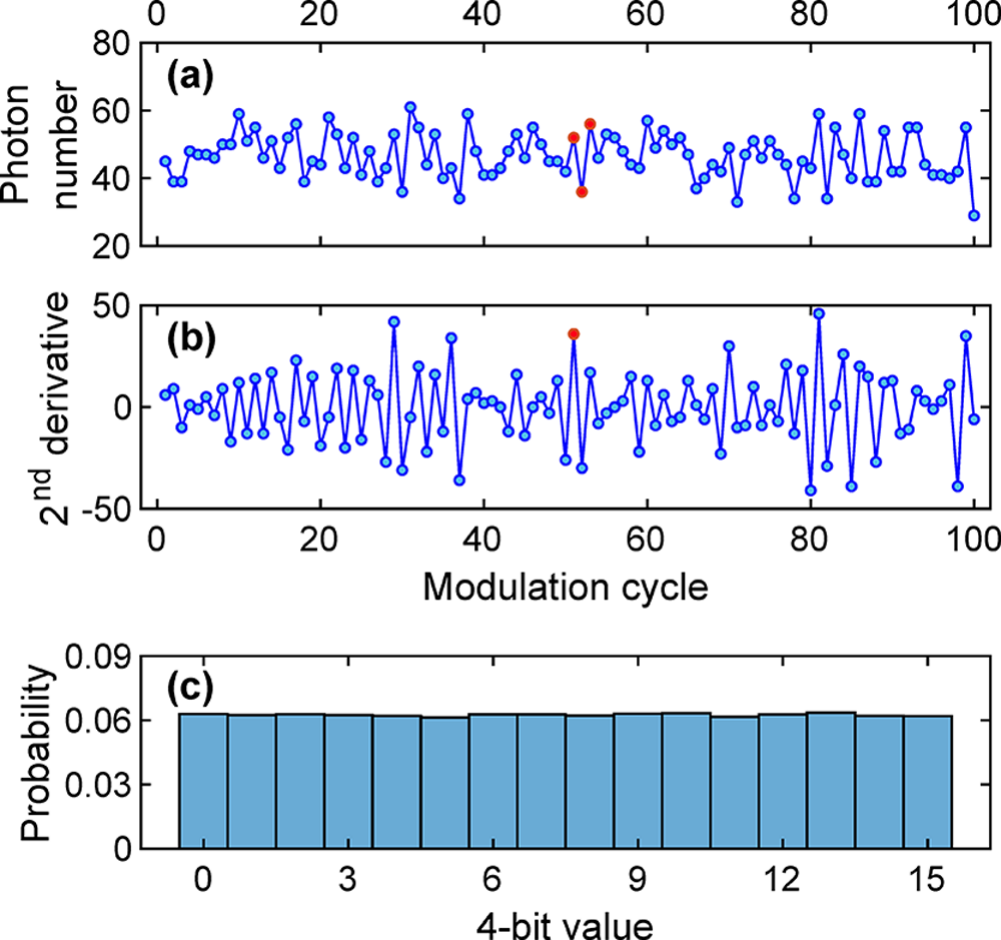
Time-series plots of (a) photon numbers derived from the voltage-time integration and (b) associated values calculated from the second derivative. (c) A histogram of 4-bit data collected from 100,000 modulation cycles.

Figure 6a presents an example of the data used for the extraction of random-bit sequences. Three connected data points (highlighted in red) were used for calculating a value of a second derivative, as presented in Figure 6b.

## 4 Results and Discussion

The statistical analysis of the 4-bit data derived from 100,000 detection events is presented in Figure 6c with a uniform probability distribution. We quantified the quantum entropy of these data with eq 2. As a result, these 4-bit data had a min-entropy of 3.9760 bits, meaning that the binary outputs had a min-entropy rate of 0.9940 per bit, which was a good indication for a high-quality noise source.

Another critical measure of a noise source involves the property being independent and identically distributed (IID). A dataset generated by a noise source is assumed IID if, and only if, each random variable in a dataset is mutually independent and has the same probability distribution. Otherwise, the dataset is non-IID, and additional postprocessing with the dataset is essential for rendering IID output before real-world use.

We followed the U.S. National Institute of Standards and Technology (NIST) Special Publication (SP) 800-90B^39^ and utilized software^40^ provided by the NIST to evaluate whether the dataset collected from the binary output is IID or not. As a result, our binary outputs passed the IID validation, suggesting that further quality improvements may not be necessary.

Next, we collected data of 160,000,000 bit-sequences from the binary outputs for statistical randomness examinations. These data were spilt into 160 datasets with equal size for validating with 15 sub-tests in accordance with the NIST SP800-22.^41^ The result, as presented in Table 1, indicates that the generated random bits passed the test suite with a significance level of 0.01.

**Table 1:** NIST SP800-22 Test Results of 160 Datasets of 1,000,000-Bit Sequences Collected from the Binary Outputs^a^

method	*P*-values-total	proportion	result
1. frequency	0.484 646	0.9938	pass
2. block frequency	0.330 628	0.9875	pass
3. runs	0.585 209	0.9938	pass
4. longest run	0.997 147	1.0000	pass
5. rank	0.701 879	0.9938	pass
6. fast Fourier transform	0.739 918	0.9875	pass
7. overlapping template	0.811 993	1.0000	pass
8. universal	0.855 534	0.9938	pass
9. linear complexity	0.934 318	1.0000	pass
10. approximate entropy	0.087 559	0.9875	pass
11. non-overlapping template	0.509 755	0.9910	pass
12. serial	0.605 729	0.9938	pass
13. cumulative sums	0.643 355	0.9938	pass
14. random excursions	0.604 063	0.9897	pass
15. random excursions variant	0.479 154	0.9908	pass

a To pass the test suite with a significance level of 0.01, the *P*-values-total and the proportion for each sub-method must be at least 0.0001 and 0.96, respectively.

**Figure 7:**
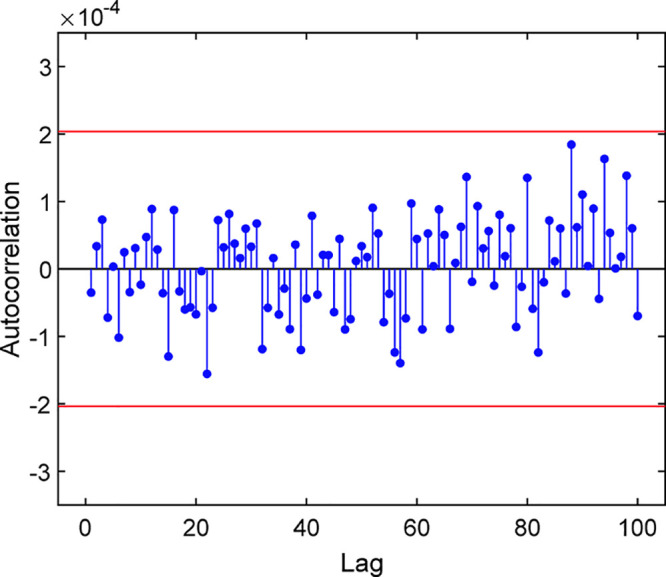
Autocorrelation (stem plot) with 99% confidence interval (red lines) calculated from 160,000,00-bit sequences of the binary outputs.

These data were also submitted to an autocorrelation analysis for quantifying the repeatability of a dataset over a time series. The result, as shown in Figure 7, indicates that autocorrelation values over 100 successive bit intervals fall within the region of the 99% confidence interval. It is assumed that a particular lag outside these two borderlines exhibits correlation with a statistical significance level of 0.01. Accordingly, it may be reasonably concluded that statistical correlations over an acceptable range were undetected from the generated random bits.

## 5 Conclusions

In summary, we proposed a scheme for the realization of QRNG based on the detection of photon numbers with the use of an SiPM. Our proposed technique implemented a time integral with the detector response signal to resolve a photon number. This scheme allows for a long pulse width of the photon flux, making it feasible to implement the system with a low-cost laser diode.

Here, the experimental proof-of-concept was simplified with the use of a budget oscilloscope for data acquisition. A computer was used for data processing and statistical analyses. Our preliminary demonstration achieved data acquisition at a rate of 3.4 MHz with an extraction ratio of 4 bits per single detection event, resulting in random bit generation being realized at a rate of 13.6 Mbit/s. Our generated random bits passed the statistical randomness validation according to the NIST SP800-22 test suite, and a repeating pattern was not found from the autocorrelation analysis.

A further improvement would be the development of an electronic module, for data acquisition and random bit extraction, with integration of the following essential components: a low-noise amplifier circuit,^42^ an integrator circuit, an analog-to-digital converter, and an FPGA. While the development of SiPM technology is in progress, upgrading to a next-generation SiPM with a short-pulse-width capability and a high megapixel count would enable high-frequency operation up to 40 MHz with an extraction ratio of up to 8 bits per detection cycle. As a result, this development would allow QRNG to achieve high bitrates, potentially up to several 100 Mbit/s. To construct a random number generator that meets industrial certification, proper engineering^43^ is vital to prevent hardware failure. The successful execution of this engineering requires the incorporation of physical safeguards that can withstand security attacks like electromagnetic wave injection.^44^ Besides, implementation of a health-monitoring scheme that complies with the NIST SP800-90B^39^ or AIS 20/31^45^ (AIS: Application Notes and Interpretation of the Scheme issued by the German Federal Office for Information Security, also known as BSI) recommendations would be mandatory to provide provable robustness against security attacks.

## Data Availability

The data that support the findings of this study are available upon reasonable request from the authors.
